# Local Neuronal Activity and the Hippocampal Functional Network Can Predict the Recovery of Consciousness in Individuals With Acute Disorders of Consciousness Caused by Neurological Injury

**DOI:** 10.1111/cns.70108

**Published:** 2024-11-07

**Authors:** Xi Wang, Xingdong Liu, Lin Zhao, Zhiyan Shen, Kemeng Gao, Yu Wang, Danjing Yu, Lin Yang, Ying Wang, Yongping You, Jing Ji, Jiu Chen, Wei Yan

**Affiliations:** ^1^ Department of Neurosurgery The First Affiliated Hospital of Nanjing Medical University Nanjing China; ^2^ Department of Nuclear Medicine The First Affiliated Hospital of Nanjing Medical University Nanjing China; ^3^ Department of Radiology, Nanjing Drum Tower Hospital, Affiliated Hospital of Medical School Nanjing University Nanjing China

**Keywords:** acute disorders of consciousness, amplitude of low‐frequency fluctuations, hippocampal functional network, recovery of consciousness, resting‐state functional MRI

## Abstract

**Aims:**

There is limited research on predicting the recovery of consciousness in patients with acute disorders of consciousness (aDOC). The purpose of this study is to investigate the altered characteristics of the local neuronal activity indicated by the amplitude of low‐frequency fluctuations (ALFF) and functional connectivity (FC) of the hippocampus network in patients with aDOC caused by neurological injury and to explore whether these characteristics can predict the recovery of consciousness.

**Methods:**

Thirty‐seven patients with aDOC were included, all of whom completed resting‐state functional magnetic resonance imaging (rsfMRI) scans. The patients were divided into two groups based on prognosis of consciousness recovery, 24 patients were in prolonged disorders of consciousness (pDOC) and 13 in emergence from minimally conscious state (eMCS) at 3 months after neurological injury. Univariable and multivariate logistic regression analyses were used to investigate the clinical indicators affecting patients' recovery of consciousness. The ALFF values and FC of the hippocampal network were compared between patients with pDOC and those with eMCS. Additionally, we employed the support vector machine (SVM) method to construct a predictive model for prognosis of consciousness based on the ALFF and FC values of the aforementioned differential brain regions. The accuracy (ACC), area under the curve (AUC), sensitivity, and specificity were used to evaluate the efficacy of the model.

**Results:**

The FOUR score at onset and the length of mechanical ventilation (MV) were found to be significant influential factors for patients who recovered to eMCS at 3 months after onset. Patients who improved to eMCS showed significantly increased ALFF values in the right calcarine gyrus, left lingual gyrus, right middle temporal gyrus, and right precuneus compared to patients in a state of pDOC. Furthermore, significant increases in FC values of the hippocampal network were observed in the eMCS group, primarily involving the right lingual gyrus and bilateral precuneus, compared to the pDOC group. The predictive model constructed using ALFF alone or ALFF combined with FC values from the aforementioned brain regions demonstrated high accuracies of 83.78% and 81.08%, respectively, with AUCs of 95% and 94%, sensitivities of 0.92 for both models, and specificities of 0.92 for both models in predicting the recovery of consciousness in patients with aDOC.

**Conclusion:**

The present findings demonstrate significant differences in the local ALFF and FC values of the hippocampus network between different prognostic groups of patients with aDOC. The constructed predictive model, which incorporates ALFF and FC values, has the potential to provide valuable insights for clinical decision‐making and identifying potential targets for early intervention.

## Graphical abstract

Local neuronal activity and the hippocampal functional network of rsfMRI can predict the recovery of consciousness at 3 months post‐onset in individuals with aDOC caused by neurological injury.

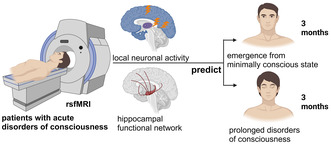

## 1 Introduction

Neurological injury diseases, including stroke and traumatic brain injury (TBI), have a high incidence rate [1, 2]. Due to the rapid development of modern emergency medical treatment of neurological injury, the percentage of patients who die from neurological injury has decreased, but the prevalence of acute disorders of consciousness (aDOC) has substantially increased, which often develop various neurological functional impairments, including prolonged disorders of consciousness (pDOC) [3, 4]. Patients with pDOC often require extended care and treatment to sustain life, incurring substantial costs that place a significant burden on society. Additionally, this situation imposes considerable emotional strain and substantial financial pressure on the patients' families, while also giving rise to a host of ethical, legal, and other related issues [5]. In clinical practice, early accurate prognostication of neurological outcomes has important guiding significance for clinical treatment, medical decision‐making and facilitates communication with family members. However, improving the ability of clinicians to reliably evaluate and prognosticate for neurological injury patients with aDOC is currently a highly concerning issue and remains a persistent challenge.

Prognostic performance of single electroencephalography (EEG) or clinical behavioral assessment in the recovery of consciousness in patients at the acute stage of disorders of consciousness (DOC) is insufficient [6, 7, 8]. The insufficient understanding of the critical brain structures and functions that lead to consciousness disorders as well as their impact on consciousness recovery is a significant factor contributing to the current lack of accurate predictive tools for assessing the recovery of consciousness in patients with aDOC. Therefore, to identify the key brain regions influencing consciousness recovery and analyze their functional activities, it is necessary to integrate or apply high spatial resolution imaging techniques capable of reflecting the functional activities and functional connectivity (FC) of brain regions. Fludeoxyglucose positron emission tomography (FDG‐PET) can evaluate the brain metabolism; functional magnetic resonance imaging (fMRI) is able to detect brain activity by measuring the blood oxygen level dependent (BOLD) signal; and diffusion tensor imaging (DTI) is capable of evaluating brain connection [9, 10, 11]. In fMRI, resting‐state functional MRI (rsfMRI) is more suitable for examining patients with impaired consciousness than task‐based fMRI, as it does not require the subject to receive or complete a specified task [12]. FMRI is able to characterize and differentiate the brain's functional status through computational approaches [13]. And the amplitude of low‐frequency fluctuation (ALFF), fractional ALFF (fALFF), and regional homogeneity (ReHo) of rsfMRI BOLD signals can be used to characterize regional neural activity [14]. By correlating the BOLD signal within regions of interest (ROIs) with all other voxels in the brain, it can generate whole‐brain FC maps of specific regions or networks, thereby reflecting the precise activity state of the brain [15]. Previous studies have investigated the diagnostic precision and predictive prognostic value of positron emission tomography (PET) imaging, fMRI, and EEG in pDOC [16, 17, 18], while precious few research have been done on aDOC.

We submitted and obtained approval from the Ethics Committee of the First Affiliated Hospital of Nanjing Medical University (NO.2023‐SR‐860, NO.2022‐SR‐354, NO.2023‐SR‐378) to collect or acquire PET/rsfMRI, EEG, and multiomics profiling of cerebrospinal fluid in neurological injury patients with aDOC and attempted to analyze the relationship between one or multiple of these indicators and the prognosis of the patients. For the current analyses, we used the BOLD date of 37 patients who had been enrolled, completed follow‐up, and qualified for BOLD imaging quality to explore the correction between BOLD features and consciousness recovery in neurological injury patients with aDOC. Previous studies have reported that there exist differences in the EEG activities of patients with aDOC, and the visual‐graded EEG is correlated with the prognosis of patients [19]. Furthermore, previous studies have found the variations in cerebral blood flow among patients with aDOC [20], while cerebral blood flow is correlated with neuronal activity and FC [21, 22]. We hypothesized that there are differences in local neuronal activity and FC during the acute phase among patients with aDOC of different prognoses. The aim of our study is to identify the altered imaging characteristics, explore their potential for early prediction of consciousness recovery, and provide a basis for future early intervention therapies to improve the prognosis of patients with aDOC.

## 2 Methods

### 2.1 Study Participants

A total of 192 neurological injury patients admitted to the neurosurgery intensive care unit (NICU) and the First Affiliated Hospital with Nanjing Medical University from September 2022 to July 2023 were recruited for the study. The inclusion criteria were as follows: (1) aged ≥ 18 years; (2) the score of Glasgow Coma Scale (GCS) at admission was ≤ 12; (3) the score of GCS before PET/rsfMRI scan was ≤ 12; (4) vital signs were stable at the time of PET/rsfMRI; (5) out of the ventilator at the time of PET/rsfMRI; (6) less than 28 days from onset of disturbance of consciousness to PET/rsfMRI scan; (7) caregivers of patients agreed to participate in the study and agreed to receive standardized hyperbaric oxygen therapy as well as multisensory stimulation for more than 1 month after leaving the NICU. The exclusion criteria were as follows: (1) past medical history of TBI, stoke, brain tumor, and hydrocephalus; (2) unstable vital signs; (3) epileptic status; (4) uncontrolled intracranial infection; (5) death before PET/rsfMRI scan; (6) GCS score > 12 during hospitalization; (7) death during follow‐up; (8) secondary hydrocephalus without timely shunt surgery; (9) received median nerve electrical stimulation or transcranial magnetic stimulation during hospitalization and follow‐up; (10) caregivers of patients refused to be enrolled or decided to withdraw them from the study at any time during the follow‐up period; and (11) BOLD date of PET/rsfMRI was unqualified.

### 2.2 Assessment of Consciousness State

The enrolled neurological injury patients with aDOC were followed up for 3 months after the onset. The consciousness state of each respondent was assessed using the coma recovery scale‐revised (CRS‐R) [23]. All patients underwent repeated CRS‐R scoring (at least 5 times within 10 days) at the follow‐up time phase, the scale was applied by trained professional raters in the presence of patients' family members and took the highest score to assess the consciousness level of the patients. According to the degree of consciousness recovery, these patients were divided into pDOC (including unresponsive wakefulness syndrome and minimally conscious state (MCS)) and emergence from minimally conscious state (eMCS).

### 2.3 Neuroimaging Data Acquisition

Resting‐state MRI data were acquired using a whole‐body 3T GE Signa PET/MR scanner (GE Healthcare, Waukesha, Wisconsin, USA) with a 19‐channel receive head‐ neck coil. Metal objects that can be removed from the patient's body were removed and foam padding and earplugs were used to limit head movement and reduce the effect of noise on participants.

Each subject first underwent an anatomical scan using a three‐dimensional T1‐weighted brain volume imaging (BRAVO) sequences, with the following parameters: axial slicing, repetition time (TR) = 8.7 ms, echo time (TE) = 3.3 ms, inversion time (TI) = 450 ms, number of slices = 158, thickness = 1.0 mm, gap = 0 mm, matrix = 256 × 256, flip angle (FA) = 12°, field of view (FOV) = 220 mm × 220 mm, voxel size = 0.9 × 0.9 × 0.9 mm^3^, number of excitation time (NEX) = 1, scan duration = 4.85 min.

A gradient echo‐echo planar imaging (GRE‐EPI) was used to collect BOLD rsfMRI and the scanning parameters were as follows: TR = 3000 ms, TE = 30 ms, FA = 90°, number of slices = 36, thickness = 4.0 mm, gap = 0 mm, matrix = 64 × 64 mm, FOV = 240 mm × 240 mm, acquisition bandwidth = 250 kHz, voxel size = 3.8 × 3.8 × 4 mm^3^, scan duration = 10.5 min.

### 2.4 Pre‐Processing of BOLD Date

Data preprocessing was carried out by toolkits of DPARSF (http://www.rfmri.org/DPARSF) and Statistical Parametric Mapping 12(SPM12) (http://www.fil.ion.ucl.ac.uk/spm) on a MATLAB (MathWorks, Natick, MA, USA) platform [24]. In the first place, the first 10 volumes of the scanning session were discarded to allow for T1 equilibration effects and slice‐time correction was performed on the remaining images. In order to reduce the motion effects on our data, we only included subjects characterized by motion parameters smaller than 3 mm translation and 3° rotation [25]. Second, harmful covariate regression was performed including linear trend, cerebral spinal fluid signals, white matter signals, and Friston‐24 parameters of head motions. Modulated images were normalized to the Montreal Neurological Institute (MNI) space by Diffeomorphic Anatomical Registration Through Exponentiated Lie algebra (DARTEL) with a resampled voxel size of 3 × 3 × 3 mm^3^. Finally, data were filtered using the low‐frequency band (0.01–0.1 Hz) and functional images were spatially smoothed with an 8‐mm full width at half maximum (FWHM).

### 2.5 Calculation and Analysis of ALFF

The ALFF is a neuroimaging measure that quantifies the magnitude of spontaneous fluctuations in the BOLD signal within low‐frequency ranges. We used DPABI to calculate the ALFF values after the image preprocessing procedure. In brief, the time series were transformed into frequency domain to obtain the power spectrum using a fast Fourier transform (FFT). The square root was then calculated and averaged across frequency intervals that were predefined. The averaged square root obtained by the procedure was considered as ALFF. To enhance the normality and reliability of the data, ALFF was then converted to a z‐normalized ALFF (zALFF) by subtracting the global mean value and dividing it by the SD [26, 27]. The resulting zALFF values were used as the final ALFF values for subsequent statistical analysis.

### 2.6 Hippocampal FC Analyses

In present study, left hippocampal region was selected as the ROI. We obtained the left hippocampus ROI using automated anatomical labeling implemented through WFU PickAtlas software [28]. For each participant, the average time courses for all voxels within the hippocampus were calculated. A voxel‐wise cross‐correlation analysis was then performed between the averaged ROI signal time course and the time series of other voxel in the remainder of the whole brain. Finally, Fisher's *z*‐transform analysis was performed to increase the normality of the correlation coefficients.

### 2.7 Statistical Analysis

Statistical analysis was performed using SPSS 23.0. In clinical characteristics, categorical variables were expressed as number of cases (*n*) and percentage (%). The normality of all continuous variables was examined by the Shapiro–Wilk test, continuous variables in a normal distribution were expressed as mean ± standard deviation ($\bar{X}$ ± SD); otherwise, they were expressed as median (M) and interquartile boundary values (P25, P75). We grouped the patients based on the outcome indicators and conducted univariable and multivariate logistic regression analyses between the groups. The variables incorporated into the analysis comprise: age, gender, etiology of aDOC, history of hypertension, history of diabetes, history of hyperlipidemia, GCS score at onset, FOUR score at onset, GCS score before scan, FOUR score before scan, cerebral hernia, subarachnoid hemorrhage (SAH), brain stem hematoma, thalamic hematoma, intracranial infection, secondary cerebral infarction, chronic hydrocephalus, length of stay in ICU, length of mechanical ventilation (MV), and days between scan and onset. Nomograms were created using an R package by incorporating variables with a *p*‐value of <0.05 identified using the multivariable logistic regression analysis.

The DPABI software was used to compare differences in ALFF indices and hippocampal FC among the two groups. To investigate the differences in ALFF at the group level, two‐sample *T*‐tests were performed on the individual ALFF maps between different states of consciousness recovery. The brain regions that reached a significant level of significance were visualized using BrainNet Viewer for brain mapping. A threshold‐free cluster enhancement (TFCE) was applied to correct the results to a significant level of *p* < 0.005, with a voxel number of ≥ 30. To investigate the differences in hippocampal FC at the group level, two‐sample *T*‐tests were performed between different states of consciousness recovery. A TFCE was applied to correct the results to a significant level of *p* < 0.005, with a voxel number of ≥ 20.

Furthermore, to investigate the prediction performance of regional ALFF values and hippocampal network, we made receiver operating characteristic (ROC) curves and calculated areas under the curve (AUCs). In summary, prediction models were built using the MATLAB platform's built‐in support vector machine (SVM) code. Feature extraction was performed using the ALFF values and hippocampal FC values in differential brain regions obtained through two‐sample *T*‐tests. Following feature dimensionality reduction, the models were constructed and cross‐validated using leave‐one‐out method to calculate prediction accuracy (ACC), sensitivity, specificity, and AUC as measures of model performance.

## 3 Result

### 3.1 Clinical Characteristics of Subjects

**FIGURE 1:**
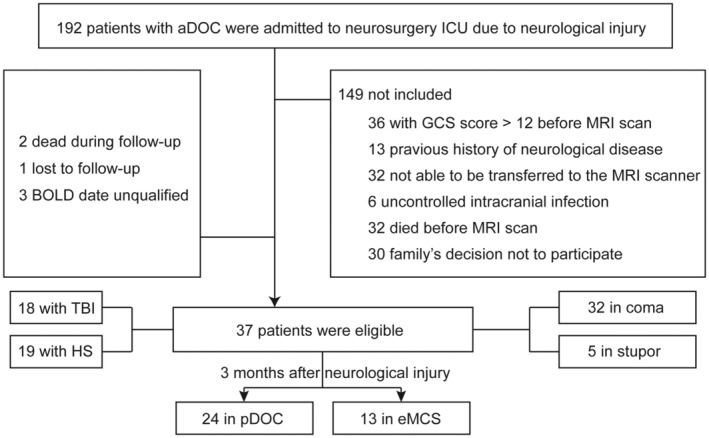
Description of the enrollment of patients in the study.

A total of 192 patients with aDOC (GCS ≤ 12 at onset) were recruited. After excluding 149 subjects that did not meet the inclusion criteria and did not undergo pet/rsfMRI measurement, 2 dead during follow‐up, 1 lost to follow‐up, and 3 subjects of BOLD date unqualified, 37 eligible patients were finally included in this study. Of the 149 excluded subjects, 36 were excluded for having a GCS score higher than 12 before MRI scan, 13 due to a previous history of neurological disease, 32 because they were not able to be transferred to the MRI scanner due to their medical condition, including unstable vital signs, epileptic status, or being unable to discontinue ventilator support, 6 due to uncontrolled intracranial infection, 32 died before MRI scan, and 30 due to the family's decision not to participate (Figure 1). The average age of the enrolled patients was 60.8 years, and 21 (56.8%) were male. Eighteen patients (48.6%) were admitted with TBI and 19 (51.4%) with hemorrhagic stroke (HS). Among them, 32 were in coma (GCS ≤ 8) at onset and 5 were in stupor (GCS 9–12) at onset. In regard to the state of consciousness recovery, 24 patients were in pDOC (64.9%) and 13 in eMCS (35.1%) at 3 months after neurological injury.

### 3.2 Independent Clinically Influential Factors for Consciousness Recovery After 3 Months of Neurological Injury With aDOC

**FIGURE 2:**
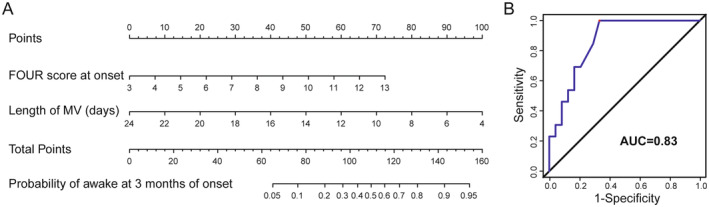
Nomogram for predicting consciousness recovery after 3 months of neurological injury with aDOC using clinical parameters. (A) To determine the probability of awake at 3 months, locate the patient's FOUR score at onset and length of MV, then draw a line straight up to the point's axis to establish the score, separately. Add the scores for each covariate together and locate the total score on the total point's axis. Draw a straight line down to the lowest line to find patient's probability of awake at 3 months. (B) ROC curves to validate the discrimination of the nomogram predicting consciousness recovery at 3 months after neurological injury with aDOC. AUC, area under the curve; FOUR, full outline of unresponsiveness; MV, mechanical ventilation.

The univariable logistic regression analysis revealed that GCS score at onset (*p* = 0.016), full outline of unresponsiveness (FOUR) score at onset (*p* = 0.011), GCS score before scan (*p* = 0.006), FOUR score before scan (*p* = 0.006), and length of MV (*p* = 0.018) were significantly correlated with consciousness statement after 3 months of neurological injury. Before multivariate logistic regressive analysis, the variance inflation factor (VIF) is employed to investigate the existence of multicollinearity between the significant variables in univariate analysis (*p* < 0.05). And the result showed that the existence of multicollinearity was among GCS score at onset, FOUR score at onset, GCS score before scan, and FOUR score before scan (VIF > 5). Due to differences in time interval between MRI scan and onset of neurological injury, the clinical significance of behavioral scale score before MRI scan is relatively low. Meanwhile, the FOUR score is better than the GCS score in responding to neurological reactions in patients with aDOC [29, 30, 31]. Therefore, we chose to retain FOUR score at onset among these four variables and conduct multivariate logistic regression analysis together with length of MV. It revealed that FOUR score at onset (odds ratio [OR], 1.613; 95% confidence interval [CI], 1.062–2.449; *p* = 0.025) and length of MV (OR, 0.719; 95% CI, 0.530–0.975; *p* = 0.034) were significant influential factors of patients recovered to eMCS at 3 months of onset (Table 1). A nomogram was then created based on influential factors identified using the multivariable logistic regression analysis to predict consciousness recovery after 3 months of neurological injury with aDOC. The AUC of the nomogram was 0.83, the predictive performance is acceptable but not excellent enough (Figure 2).

**TABLE 1:** Univariable and multivariable logistic regression analyses on demographics, clinical, and surgical characteristics of pDOC and eMCS subjects.

Items	pDOC	eMCS	Univariate analysis		Multivariate analysis	
	*n* = 24	*n* = 13	OR (95% CI)	*p*	OR (95% CI)	*p*
Age (years)	62.5 ± 12.3	57.7 ± 13.5	0.970 (0.919–1.024)	0.276		
Gender (male)	13 (54.2%)	8 (61.5%)	1.354 (0.342–5.360)	0.666		
Etiology of aDOC						
TBI	11 (45.8%)	7 (53.8%)	1.379 (0.356–5.341)	0.642		
HS	13 (54.2%)	6 (46.2%)				
Hypertension	15 (62.5%)	9 (69.2%)	1.350 (0.320–5.691)	0.682		
Diabetes	3 (12.5%)	1 (7.7%)	0.583 (0.054–6.251)	0.653		
Hyperlipidemia	6 (25.0%)	3 (23.1%)	0.900 (0.184–4.400)	0.896		
GCS score at onset	4 (3,5)	6 (5,10)	1.507 (1.080–2.103)	0.016		
FOUR score at onset	6.7 ± 2.7	9.3 ± 1.7	1.596 (1.114–2.287)	0.011	1.613 (1.062–2.449)	0.025
GCS score before scan	6.2 ± 2.1	8.6 ± 1.8	1.830 (1.194–2.805)	0.006		
FOUR score before scan	10 (8.3,11.8)	13 (11.5,13.0)	2.189 (1.253–3.813)	0.006		
Cerebral hernia	9 (37.5%)	1 (7.7%)	0.139 (0.015–1.255)	0.079		
SAH	16 (43.2%)	6 (46.2%)	0.429 (0.108–1.707)	0.229		
Brain stem hematoma	9 (37.5%)	1 (7.7%)	0.139 (0.015–1.255)	0.079		
Thalamic hematoma	8 (33.3%)	2 (15.4%)	0.364 (0.065–2.050)	0.252		
Intracranial infection	4 (16.7%)	4 (30.8%)	2.222 (0.452–10.937)	0.326		
Secondary cerebral infarction	12 (50.0%)	2 (15.4%)	0.182 (0.033–1.001)	0.050		
Chronic hydrocephalus	6 (25.1%)	3 (23.1%)	0.900 (0.184–4.400)	0.896		
Length of stay in ICU (days)	19.2 ± 4.8	16.8 ± 5.5	0.900 (0.774–1.047)	0.173		
Length of MV (days)	13.3 ± 3.9	9.9 ± 2.6	0.727 (0.558–0.947)	0.018	0.719 (0.530–0.975)	0.034
Days between scan and onset	16.9 ± 4.0	15.4 ± 4.4	0.909 (0.765–1.082)	0.283		

Abbreviations: eMCS, emergence from minimally conscious state; HS, hemorrhagic stroke; MV, mechanical ventilation; pDOC, prolonged disorders of consciousness; SAH, subarachnoid hemorrhage; TBI, traumatic brain injury.

### 3.3 ALFF

**FIGURE 3:**
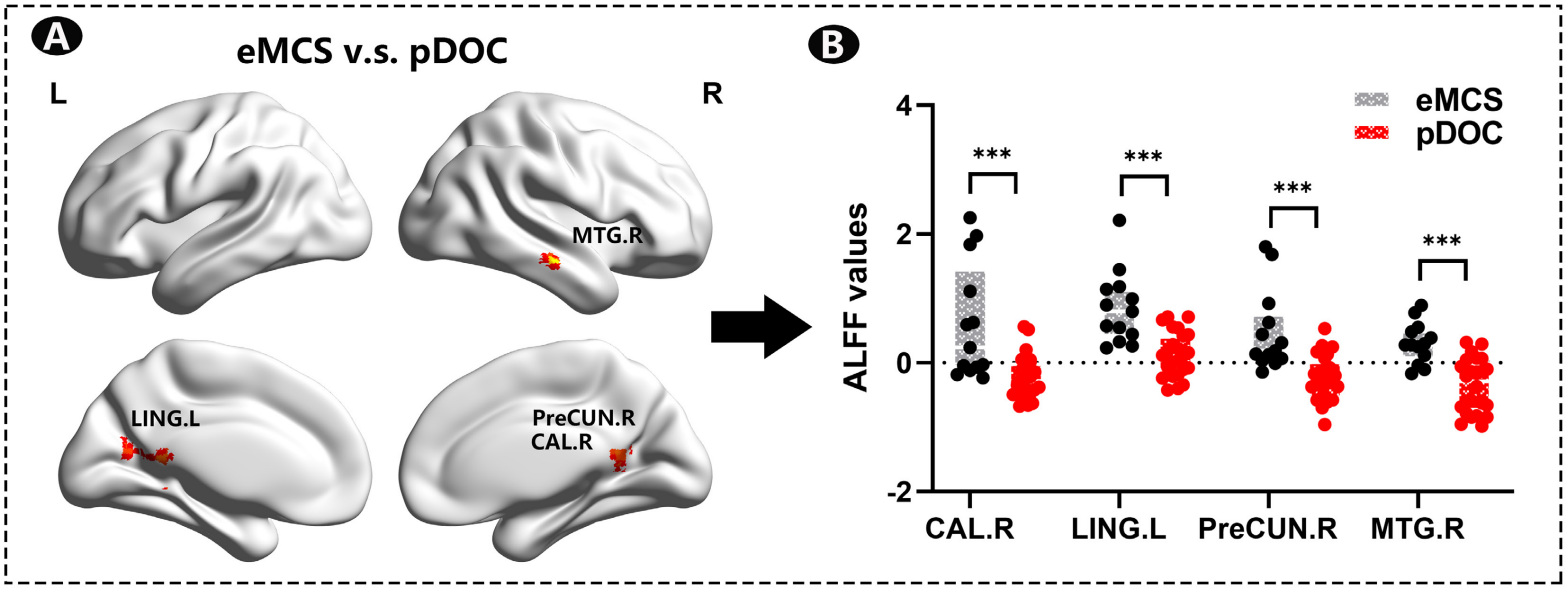
Significant differences in ALFF between pDOC and eMCS subjects. Two sample *t*‐tests between the eMCS patient group and the pDOC group in resting‐state ALFF, *p* < 0.005, voxel numbers ≥ 30, TFCE correction. (A) Red areas indicate the cerebral regions in which the ALFF of eMCS patients were significantly higher than the pDOC group. (B) The z‐normalized ALFF values in different brain regions for the eMCS and pDOC groups, respectively. ALFF, amplitude of low‐frequency fluctuation; CAL.R, right calcarine gyrus; eMCS, emergence from minimally conscious state; LING.L, left lingual gyrus; MTG.R, right middle temporal gyrus; pDOC, prolonged disorders of consciousness; preCUN.R, right precuneus; TFCE, threshold‐free cluster enhancement.

Two sample *t*‐tests showed that the brain regions with significant differences in ALFF between the patients improved to eMCS and patients in state of pDOC were mainly located in the right calcarine gyrus (CAL.R), left lingual gyrus (LING.L), right middle temporal gyrus (MTG.R), and right precuneus (preCUN.R) (*p* < 0.005, TFCE correction) (Figure 3A, Table 2). The ALFF values of the CAL.R, LING.L, MTG.R, and preCUN.R were significantly higher in the eMCS subjects than the pDOC subjects (Figure 3B).

**TABLE 2:** Comparisons of ALFF between pDOC and eMCS subjects.

Brain regions	L/R	MNI			*T*	Cluster size (mm^3^)
		*x*	*y*	*z*		
Calcarine gyrus	R	24	−54	9	3.680	30 (810)
Lingual gyrus	L	−12	−42	−9	5.058	36 (972)
Middle temporal gyrus	R	66	–18	−12	5.190	64 (1728)
Precuneus	R	18	–57	21	3.981	151 (4077)

*Note:* Regions with significant voxel numbers ≥ 30 and *p* < 0.005 with TFCE correction were presented and defined from the regions of interest (ROIs) in AAL3v1 atlas (Rolls et al. [61]).

Abbreviations: ALFF, amplitude of low frequency fluctuation; eMCS, emergence from minimally conscious state; L, left hemisphere; MNI, Montreal neurological institute; pDOC, prolonged disorders of consciousness; R, right hemisphere.

### 3.4 FC of Hippocampus Network

**FIGURE 4:**
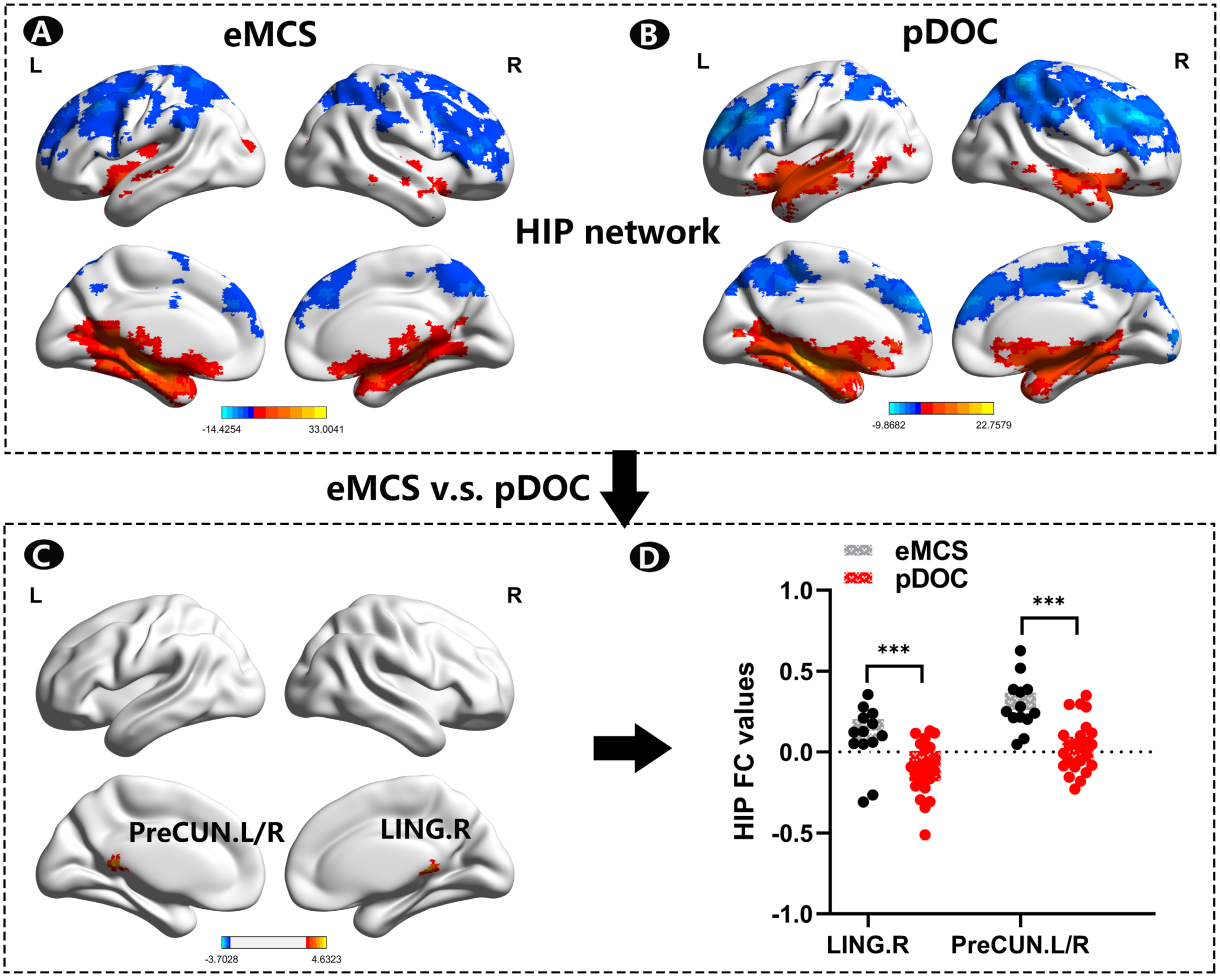
FC of hippocampus network in pDOC and eMCS subjects. (A) The FC patterns of hippocampus in eMCS subjects. (B) The FC patterns of hippocampus in pDOC subjects. (C) Two‐sample *t*‐tests between the eMCS patient group and the pDOC group in FC of the hippocampus, *p* < 0.005, voxel numbers ≥ 20, TFCE correction. Red areas indicate the cerebral regions with more noticeably increased FC of hippocampus network in eMCS patients than in the pDOC group. (D) The hippocampus network FC values in different brain regions for the eMCS and pDOC groups, respectively. eMCS, emergence from minimally conscious state; FC, functional connectivity; HIP, hippocampus; LING.R, right lingual gyrus; pDOC, prolonged disorders of consciousness; preCUN.L/R, bilateral precuneus; TFCE, threshold‐free cluster enhancement.

The FC patterns of hippocampus in eMCS subjects and pDOC subjects were estimated separately (Figure 4A,B). Two‐sample *t*‐tests were performed to investigate the differences in FC of the hippocampus between patients recovered to eMCS and patients in a state of pDOC. Significantly increased FC was observed in the eMCS group primarily involving the right lingual gyrus (LING.R) and bilateral precuneus (preCUN.L/R) than in the pDOC group (TFCE corrected *p* < 0.005) (Figure 4C,D, Table 3).

**TABLE 3:** Comparisons of HIP network between pDOC and eMCS subjects.

Brain regions	L/R	MNI			*T*	Cluster size (mm^3^)
		*x*	*y*	*z*		
Lingual Gyrus	R	3	–84	−15	3.940	21 (567)
Precuneus	R /L	−3	–45	9	4.632	89 (2403)

*Note:* Regions with significant voxel numbers ≥ 20 and *p* < 0.005 with TFCE correction were presented and defined from the regions of interest (ROIs) in AAL3v1 atlas (Rolls et al. [61]).

Abbreviations: eMCS, emergence from minimally conscious state; HIP, hippocampus; L, left hemisphere; MNI, Montreal Neurological Institute; pDOC, prolonged disorders of consciousness; R, right hemisphere.

### 3.5 Validating the Potential Predictive Value of ALFF and FC of the Hippocampal Network for Consciousness Recovery

As shown in Figure 3, significant differences in ALFF values were observed in the CAL.R, LING.L, MTG.R, and preCUN.R regions between the eMCS and pDOC groups, suggesting that these regions' ALFF values could potentially serve as markers for predicting the prognosis of consciousness recovery. Similarly, differential hippocampal FC networks are also considered in this way.

**FIGURE 5:**
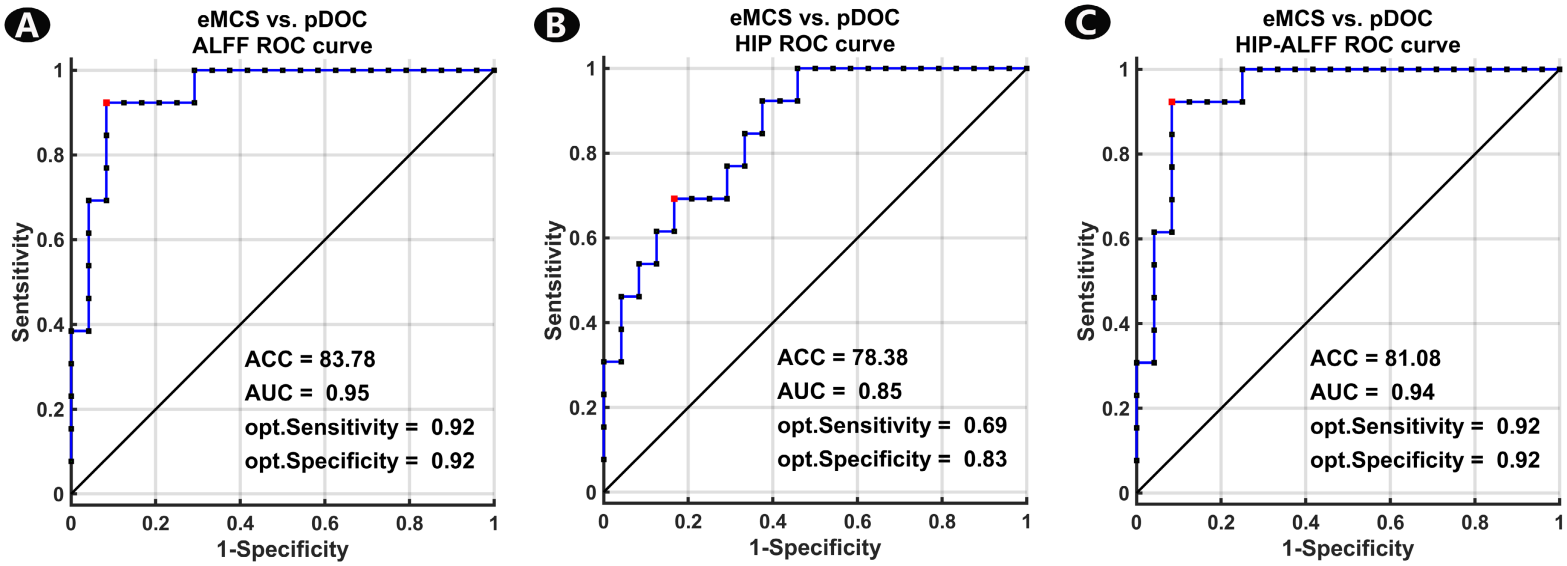
Predictive efficacy and ROC curves of eMCS vs. pDOC. (A) The predictive efficacy and ROC curves of predictive model based on the ALFF values of entire differential brain regions. (B) The predictive efficacy and ROC curves of predictive model based on the differential brain regions of hippocampal functional connectivity network. (C) The predictive efficacy and ROC curves of predictive model based on both ALFF differential brain regions and hippocampal network. ACC, accuracy; ALFF, amplitude of low‐frequency fluctuation; AUC, area under the curve; eMCS, emergence from minimally conscious state; HIP, hippocampus; pDOC, prolonged disorders of consciousness; ROC, receiver operating characteristic.

The predictive model based on the ALFF values of entire differential brain regions showed the highest efficacy in predicting the conscious state of patients with aDOC at 3 months after onset, with an ACC of 83.78, an AUC of 0.95, a sensitivity of 0.92, and a specificity of 0.92 (Figure 5A). The predictive model based on the FC values of entire differential brain regions of the hippocampal FC network achieved an ACC of 78.38 and an AUC of 0.85, a sensitivity of 0.69, and a specificity of 0.83 (Figure 5B). When combining both ALFF differential brain regions and hippocampal network, a comprehensive predictive model yielded an ACC of 81.08 and an AUC of 0.94, a sensitivity of 0.92, and a specificity of 0.92 (Figure 5C).

Furthermore, we used ALFF values of the CAL.R, LING.L, MTG.R, and preCUN.R separately to construct predictor models (Figures S1–S4) and separately used FC between LING.R, preCUN.L/R, and the hippocampus to construct predictor models (Figures S5 and S6). We calculated the ACC and AUC of these models, which were all significantly lower than the predictor model constructed by combining multiple differential brain regions.

## 4 Discussion

aDOC can be classified into coma, stupor, and hypnosis based on the level of consciousness. Among them, patients with coma and stupor have a deeper level of conscious disturbance and are more likely to develop long‐term consciousness impairment [3, 32, 33]. Our study focused on patients with acute brain injury who had a GCS score of 12 or less and aimed to identify imaging biomarkers that could predict the consciousness prognosis of coma and lethargy patients. There have been infrequent previous studies on the prognosis of consciousness recovery in patients with aDOC. Additionally, due to the critical condition of these patients and the high risk associated with conducting examinations outside of the intensive care unit, there is a scarcity of studies focusing on MRI examinations for patients with aDOC. A previous study examined the predictive value of fMRI in determining the level of consciousness in comatose patients at ICU discharge and found that DTI measurements may be possible predictors, while the BOLD measurements showed no predictive value for the state of consciousness at ICU discharge [34]. The study had a significant limitation due to the brief time interval between fMRI examination and evaluation of conscious outcomes, which could influence the ACC of the conclusion. The restoration of consciousness in many patients with aDOC requires time and rehabilitation methods [3, 35, 36]. Therefore, we considered that evaluating the prognosis of consciousness at a later stage after recurrence is more clinically meaningful. In this study, we selected the 3 months post‐onset time point for assessment. Furthermore, all the patients ultimately included in the study received hyperbaric oxygen and multisensory stimulation therapy for more than 1 month after discharge, thus mitigating potential bias resulting from significant differences in rehabilitation interventions.

We initially analyzed the clinical data that could potentially impact the recovery of consciousness in patients with acute conscious disturbance and subsequently examined the fMRI indicators influencing the prognosis of conscious recovery. This study found that the FOUR score at onset and length of MV were significant influential factors of patients recovered to eMCS at 3 months of onset. The FOUR score at onset reflected the severity of the patient's primary brain injury and the level of consciousness impairment on the day of onset. The duration of MV can serve as an indicator of the rate at which brain injury patients achieve stability in their condition and recover respiratory‐related neurological function, as meeting the necessary criteria for ventilator discontinuation includes stable underlying pathology and restoration of autonomous breathing [37]. The predictive ability of the model constructed using these two clinical indicators for the recovery of consciousness in patients with aDOC within 3 months was satisfactory but not sufficiently high. We conducted further analysis on the correlation between the fMBI indicator and consciousness prognosis.

This study used the ALFF and HIP functional network from resting‐state BOLD‐fMRI index to predict consciousness recovery in neurological injury with aDOC for the first time. In our research, we initially observed significant differences in the ALFF values of the CAL.R, LING.L, MTG.R, and preCUN.R brain regions between the pDOC and eMCS groups. Meanwhile, predictive models constructed using these brain regions accurately forecasted the recovery of consciousness in aDOC patients at 3 months post‐onset. These findings indicated that the activity intensity of these brain regions during the acute phase of neurological injury significantly influenced the outcome of consciousness recovery.

Similar to our findings, current studies generally indicate that the preCUN is a brain region closely associated with the degree of consciousness impairment and correlated with the recovery of consciousness. A study had revealed that connectivity strength in the preCUN and the posterior cingulate cortex had a significant association with recovery of neurological function in comatose cardiopulmonary arrest survivors [38]. Another study involving 2 cases of aDOC and 13 cases of pDOC, which underwent two fMRI scans at different intervals, revealed a significant correlation between longitudinal changes of ALFF values in the preCUN and the recovery of consciousness [39]. In pDOC, patients in vegetative state (VS) exhibit decreased activity in the preCUN compared to those in MCS [40, 41]. Functional connectivity analysis of fMRI in 27 VS patients revealed that the degree centrality of the preCUN was significantly higher in patients who emerged from VS compared to those who remained in VS at least 3 months after MRI scan [42]. A previous study confirmed that the preCUN plays an important role in multisensory information integration [43], suggesting that patients with enhanced preCUN activity may benefit more from multisensory stimulation therapy during the rehabilitation phase compared to patients with low preCUN activity.

In the research on brain targets of intervention measures to improve consciousness, Ma et al. [44] reported that pDOC patients treated with trigeminal nerve stimulation showed significant increases in metabolism in the right para‐hippocampal cortex (PHC.R), preCUN.R and bilateral middle cingulate cortex (MCC.L/R), accompanied by marked improvements in their consciousness state. However, there has been no research so far on stimulating the preCUN to promote consciousness recovery during the acute stage of DOC.

In the current findings, MTG.R showed closely relation to the prognosis of consciousness statement of aDOC patients. This finding is similar to previous literature, which showed that the left middle temporal gyrus (MTG.L) was one of the critical nodes within the brain's global functional network that support consciousness, whose degree centrality was significantly reduced when consciousness was reduced or absent [45]. The study revealed that one of the key brain regions supporting consciousness is located in the MTG.L, while our research identified one of the key brain regions associated with improved consciousness as being situated in the MTG.R. This lateral asymmetry may be related to individual differences among the subjects included in the studies. Although research on the recovery of aDOC is limited, the difference between the non‐lucid dreams and the lucid dreams can be analogized to the difference between aDOC and emerged from aDOC. A previous study found that the anterior prefrontal cortex (aPFC)–angular gyrus (AG)–MTG network is significantly activated in lucid dreams compared to non‐lucid dreams, suggesting that the neural circuitry involving MTG enabling the integration between heteromodal metacognitive and linguistic/conceptual systems, allowing people to be aware of oneself and one's current state and transform primary consciousness in sleep to higher‐level consciousness [46].

Our study also revealed significant differences in activity levels of CAL.R and LING.L among different consciousness recovery groups, while previous studies have not reported any relationship between these two brain regions and consciousness or disorders of consciousness. However, the calcarine gyrus (CAL) and the lingual gyrus (LING) share a commonality in that they are both closely associated with cognitive functions. In patients with phenylketonuria, a decrease in the thickness of the LING.L is correlated with worse cognitive performance [47]. In patients with idiopathic normal pressure hydrocephalus, reduced cerebral blood flow of LING is associated with cognitive impairment [48]. Moreover, previous researches have reported that the activity in the CAL is significantly lower in patients with cognitive impairment due to obsessive‐compulsive disorder and autism spectrum disorder compared to the control group [49, 50]. In addition, a study has reported that transcutaneous vagus nerve stimulation can lead to spontaneous neural activity in the CAL, LING, and parahippocampal gyrus, thereby improving cognitive function in healthy adults [51]. While cognition and consciousness are distinct thinking processes, they exhibit significant interrelationships. Improving the residual cognitive function in patients with DOC can facilitate the recovery of consciousness [52, 53].

The hippocampal complex receives reportage from widely distributed structures around the brain and organizes and binds those reports together into a brand new episodic memory, which is the only unified and contextualized representation of self‐in‐the‐world in the brain, making the hippocampal a gateway to consciousness [54, 55]. At the same time, a study has found that the hippocampal memory system can operate without consciousness and is necessary for unconscious relational encoding and retrieval [56]. Therefore, we studied the hippocampal FC network in the neurological injury patients with aDOC. Interestingly, the functional connection between preCUN.L/R, LING.R, and the hippocampus was significantly higher in the eMCS group than in the pDOC group, suggesting that information exchange between preCUN.L/R, LING.R, and the hippocampus plays a positive role in promoting consciousness recovery. We hypothesize that the information transmitted through these two regions and interacting with the hippocampus may be more crucial for the maintenance and restoration of consciousness than information transmitted through other brain regions.

In this study, an SVM approach was employed to construct predictive models and access the utility of ALFF values in differentially expressed brain regions as a neuroimaging biomarker to predict the consciousness prognosis in patients with aDOC. The same method was employed to analyze differences in hippocampal FC indicators. The SVM machine learning algorithm classifies high‐dimensional data points through the maximization of margins between classes. It is frequently employed in the analysis of mental illness and neurological systems due to its high classification ACC and capacity for handling high‐dimensional data [57, 58]. Predictive models constructed using the ALFF values of the CAL.R, LING.L, MTG.R, and preCUN.R accurately forecasted the recovery of consciousness in aDOC patients at 3 months post‐onset. The predictive model constructed using the differentiated hippocampal functional network exhibited lower predictive efficacy than the model constructed by the ALFF values, but still demonstrated high predictive efficiency. Based on the prediction model, assessing neurological injury patients with aDOC who have a higher probability of awakening can facilitate the patient's family’ decision on continuing treatment. While the early prediction of a lower probability of awakening for patients can prevent some of these patients' families from investing excessive medical resources and ultimately failing to obtain the ideal outcome. These research findings also provide theoretical support and alternative intervention targets for clinicians to potentially improve prognosis by stimulating specific brain regions during the acute phase of neurological injury. In future studies, we can activate the key brain regions influencing the outcome of consciousness discovered by our research through repetitive transcranial magnetic stimulation (rTMS) or transcranial direct current stimulation (tDCS) at the stage of aDOC, thereby providing more reliable evidence for clinical intervention. Furthermore, an exciting non‐invasive brain stimulation technique has been identified in recent years that enables precise activation of deep brain target regions through temporal interference (TI) stimulation, and this technique has entered the preclinical stage [59, 60]. Once this technology or other new non‐invasive deep brain stimulation techniques are employed in clinical settings, they can all be utilized as intervention measures for our subsequent studies.

## 5 Limitations

Firstly, the main limitation is the small number of cases included in the study. Variations in the primary disease types and baseline levels of acute consciousness among the study subjects may lead to inaccuracies in the final research conclusions. Additionally, despite the statistical significance of our findings, they may not be generalizable to the entire population of patients with aDOC. Secondly, although the clinical research design involved the collection of PET, BOLD, DTI sequences, EEG data, and cerebrospinal fluid specimens from patients, this study only analyzed the predictive value of BOLD sequences for consciousness state prognosis. In future research efforts, we aim to integrate multimodal data to identify brain regions associated with consciousness recovery that hold greater clinical significance and yield more effective predictive indicators.

## 6 Conclusion

Patients suffering from aDOC that progress to pDOC pose significant challenges to public health. However, there is a lack of research in this area. Our analysis of the correlation between fMRI BOLD sequences and recovery of consciousness in these patients has revealed notable differences in ALFF within specific brain regions and hippocampal networks associated with consciousness recovery. This information has been utilized to develop an effective predictive model, which can aid clinical decision‐making and serve as a basis for exploring early interventions to enhance the prognosis of aDOC.

## Ethics Statement

Human subjects: All participants provided written informed consent from their caregivers. The study was approved by Ethics Committee of the First Affiliated Hospital of Nanjing Medical University (Nos. 2023‐SR‐860, 2022‐SR‐354, and 2023‐SR‐378), according to the Declaration of Helsinki and Good Clinical Practice guidelines.

## Conflicts of Interest

The authors declare no conflicts of interest.

## Supporting information


Figure S1

## Data Availability

The clinical and imaging datasets analyzed during the current study are available from the corresponding author upon reasonable request.
