# Supplementary material for: Local Neuronal Activity and the Hippocampal Functional Network Can Predict the Recovery of Consciousness in Individuals With Acute Disorders of Consciousness Caused by Neurological Injury
Source: CNS Neurosci Ther. 2024 Nov 7;30(11):e70108. doi: 10.1111/cns.70108 (PMC11541605; doi:10.1111/cns.70108)
Supplement: Supplementary file 1 — Figure S1 [file CNS-30-e70108-s001.docx]

**Supplement Figure**

**Figure S1.**


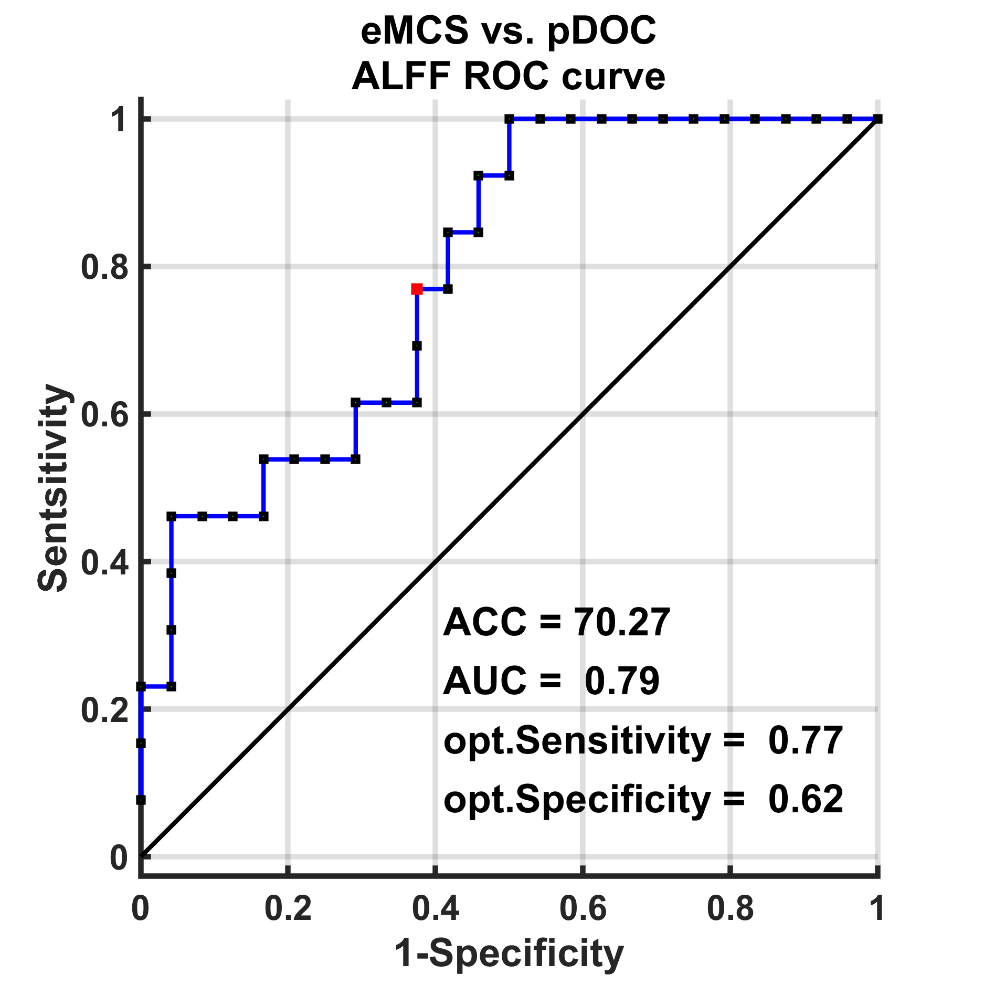


Figure S1. The predictive efficacy and ROC curves of predictive model based on the ALFF values of CAL.R.

**Figure S2.**


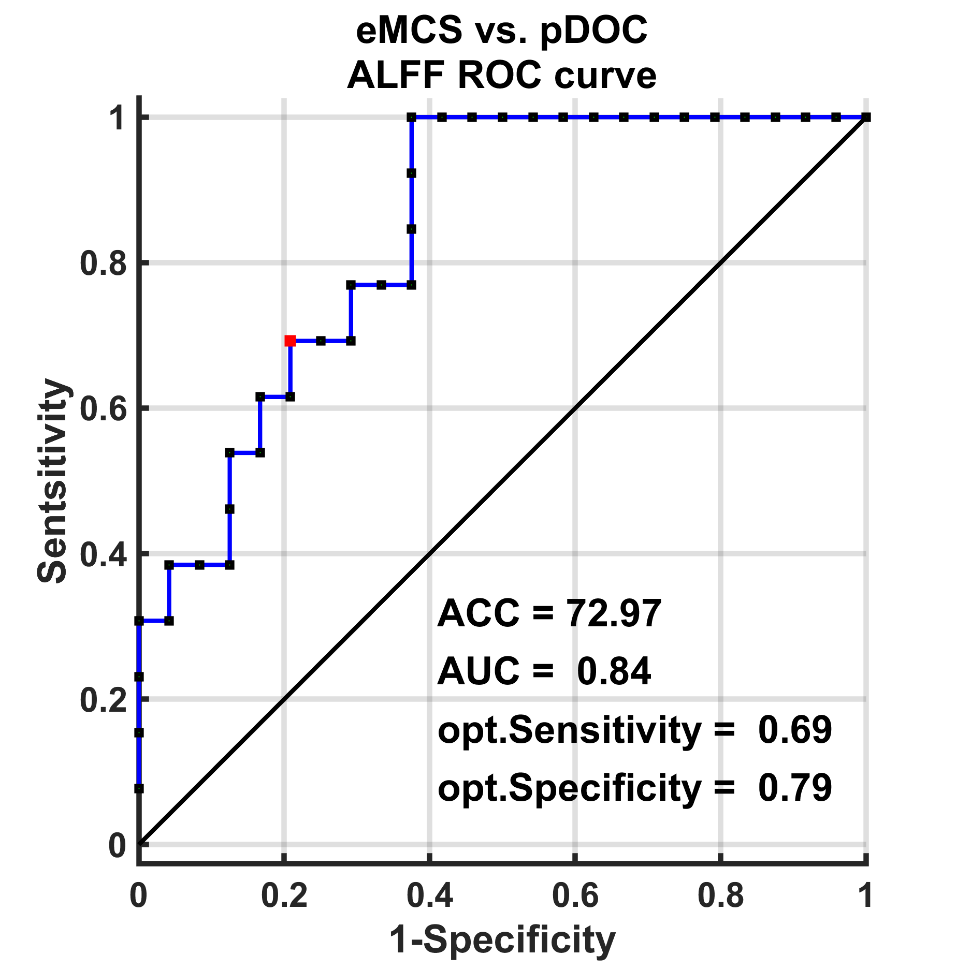


Figure S2. The predictive efficacy and ROC curves of predictive model based on the ALFF values of LING.L.

**Figure S3.**


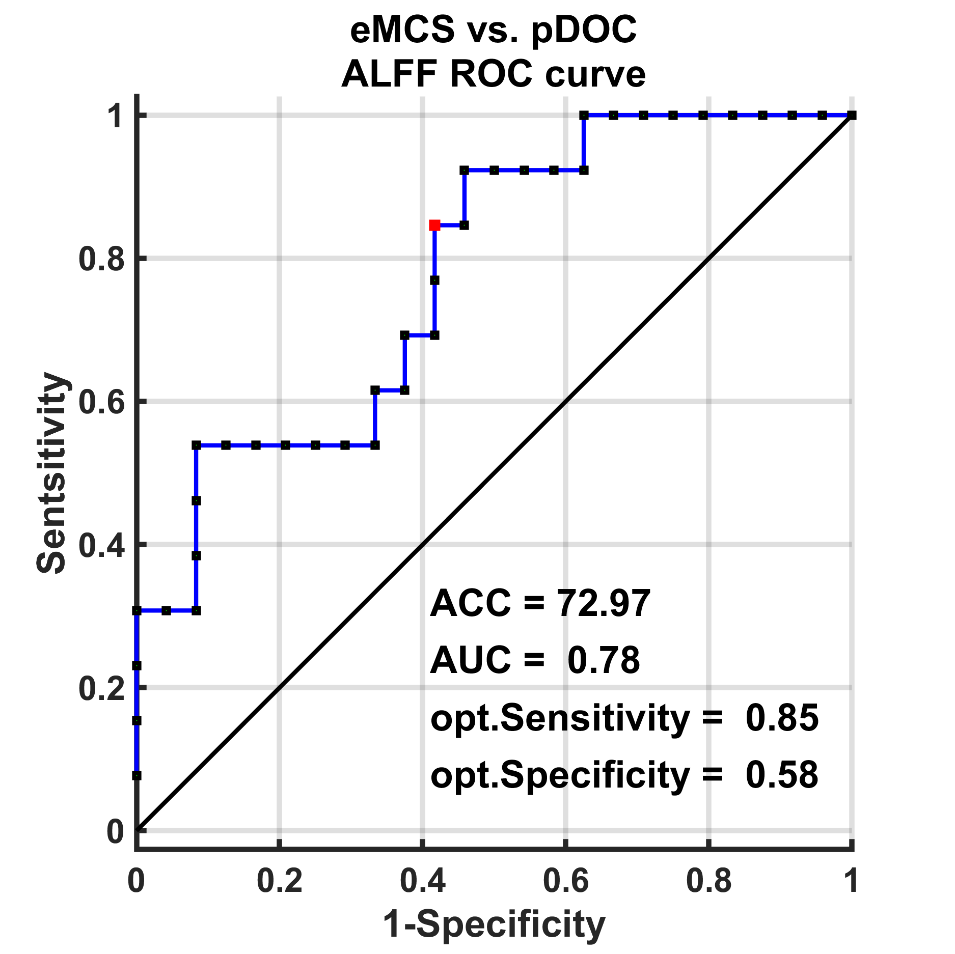


Figure S3. The predictive efficacy and ROC curves of predictive model based on the ALFF values of MTG.R.

**Figure S4.**


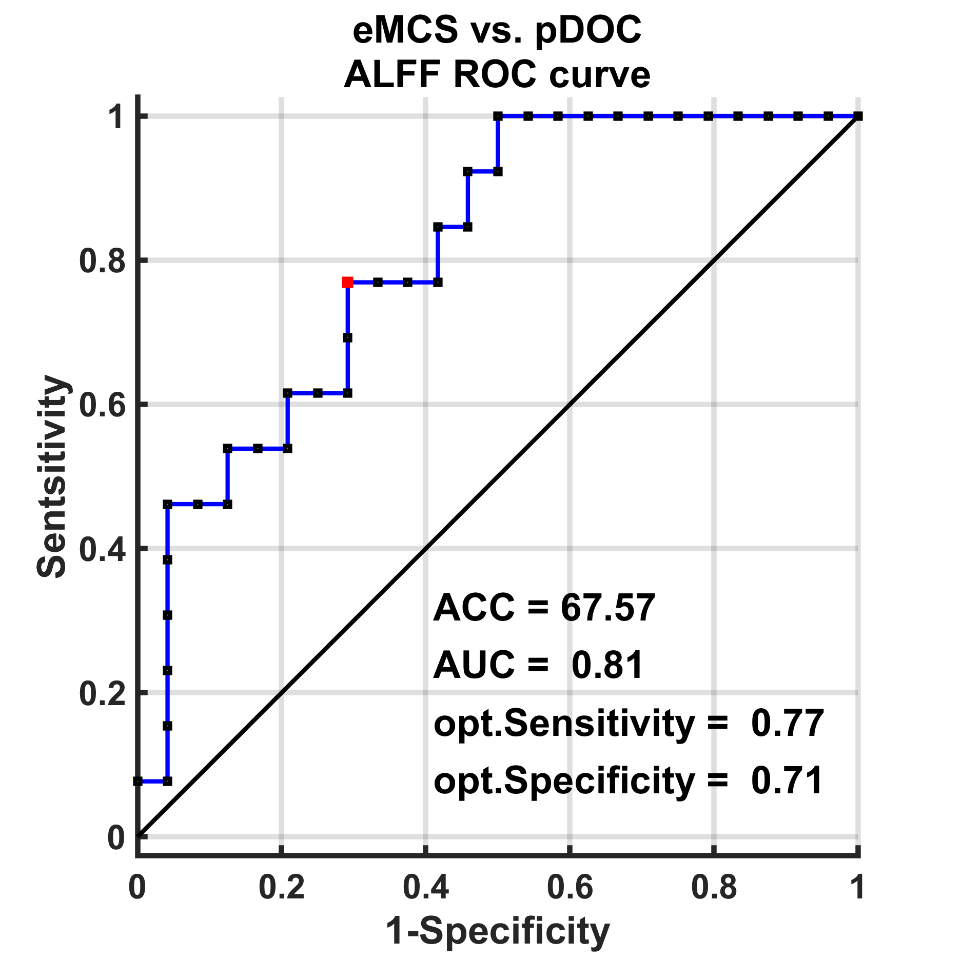


Figure S4. The predictive efficacy and ROC curves of predictive model based on the ALFF values of preCUN.R.

**Figure S5.**


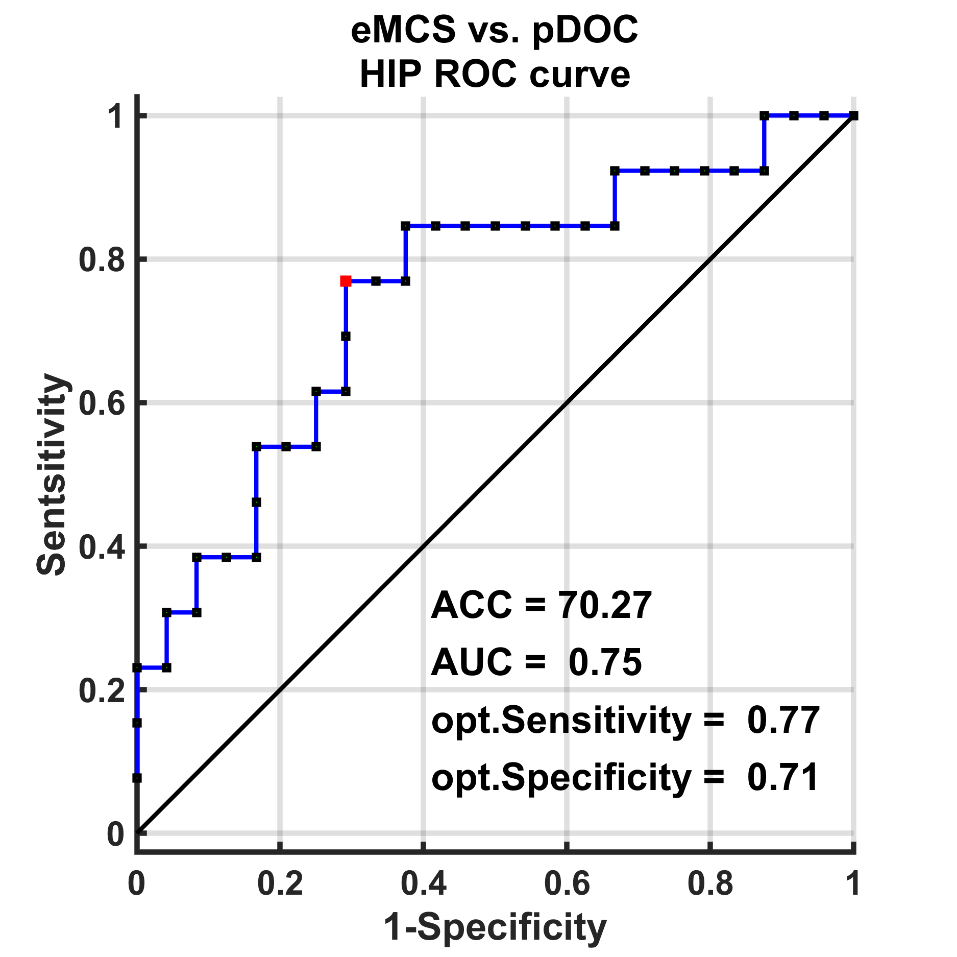


Figure S5. The predictive efficacy and ROC curves of predictive model based on the FC between LING.R and the hippocampus.

**Figure S6.**


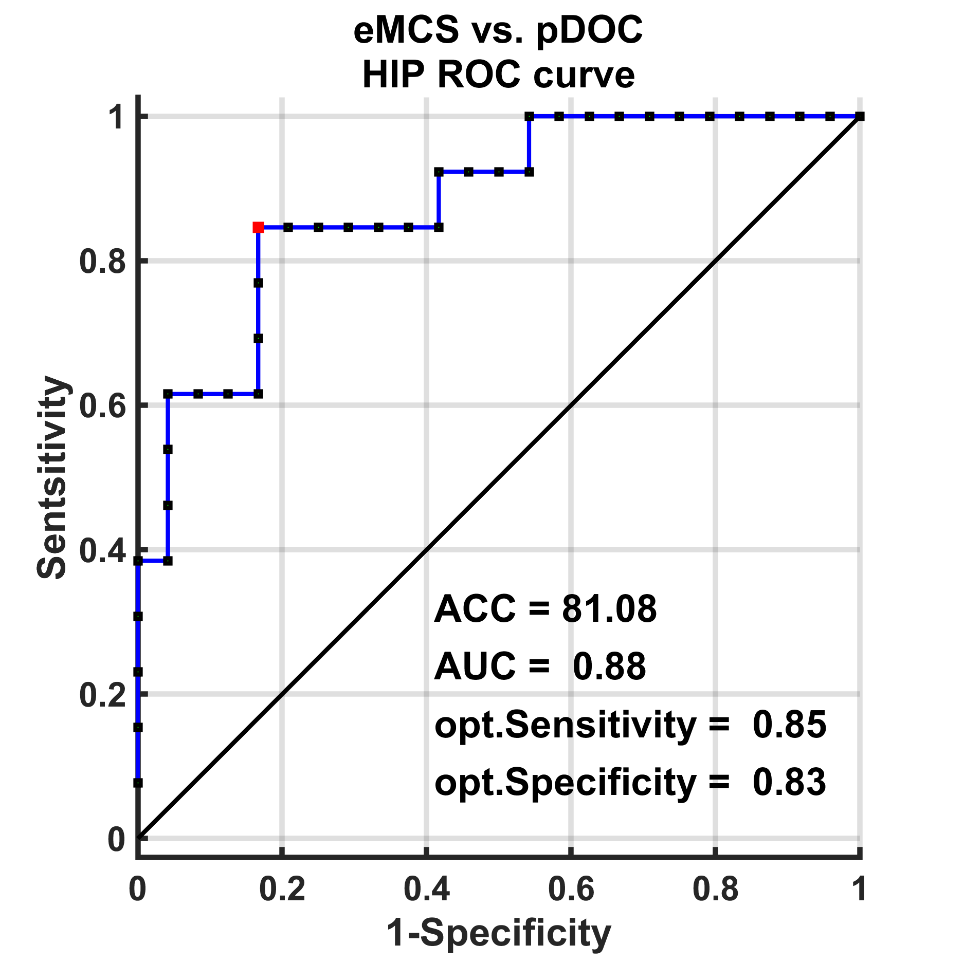


Figure S6. The predictive efficacy and ROC curves of predictive model based on the FC between preCUN.L/R and the hippocampus.
